# Supplementary material for: Social training reconfigures prediction errors to shape Self-Other boundaries
Source: Nat Commun. 2020 Jun 15;11:3030. doi: 10.1038/s41467-020-16856-8 (PMC7295766; doi:10.1038/s41467-020-16856-8)
Supplement: Supplementary file 1 — Supplementary Information [file 41467_2020_16856_MOESM1_ESM.pdf]

Supplementary Information for

Social training reconfigures prediction errors to shape Self-Other boundaries

by Ereira et al.

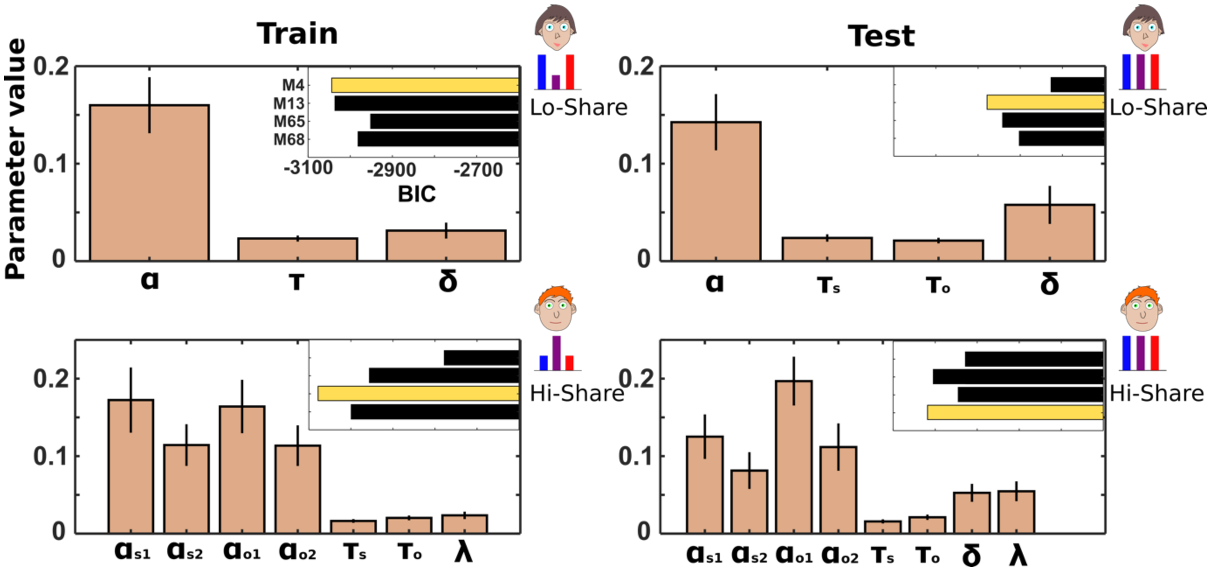

Supplementary Fig. 1 | Parameter estimates. Mean parameter estimates are shown for each of the winning models fit to the four FBT datasets (Hi-Share and Lo-Share in training and Hi-Share and Lo-Share in testing). Insets show the relative model evidences (Bayesian information criterion (BIC) summed across subjects) of these four models with the winning model highlighted in yellow. Parameter subscript letters (s/o) denote whether the parameter is specific to Self-updates or Other-updates. Parameter subscript numbers (1/2) denote whether the learning rate is for single agent updates ('privileged' and 'decoy' trials) or double agent updates ('shared' trials). Error bars denote s.e.m. n = 40 independent subjects. Source data are provided as a Source Data file.

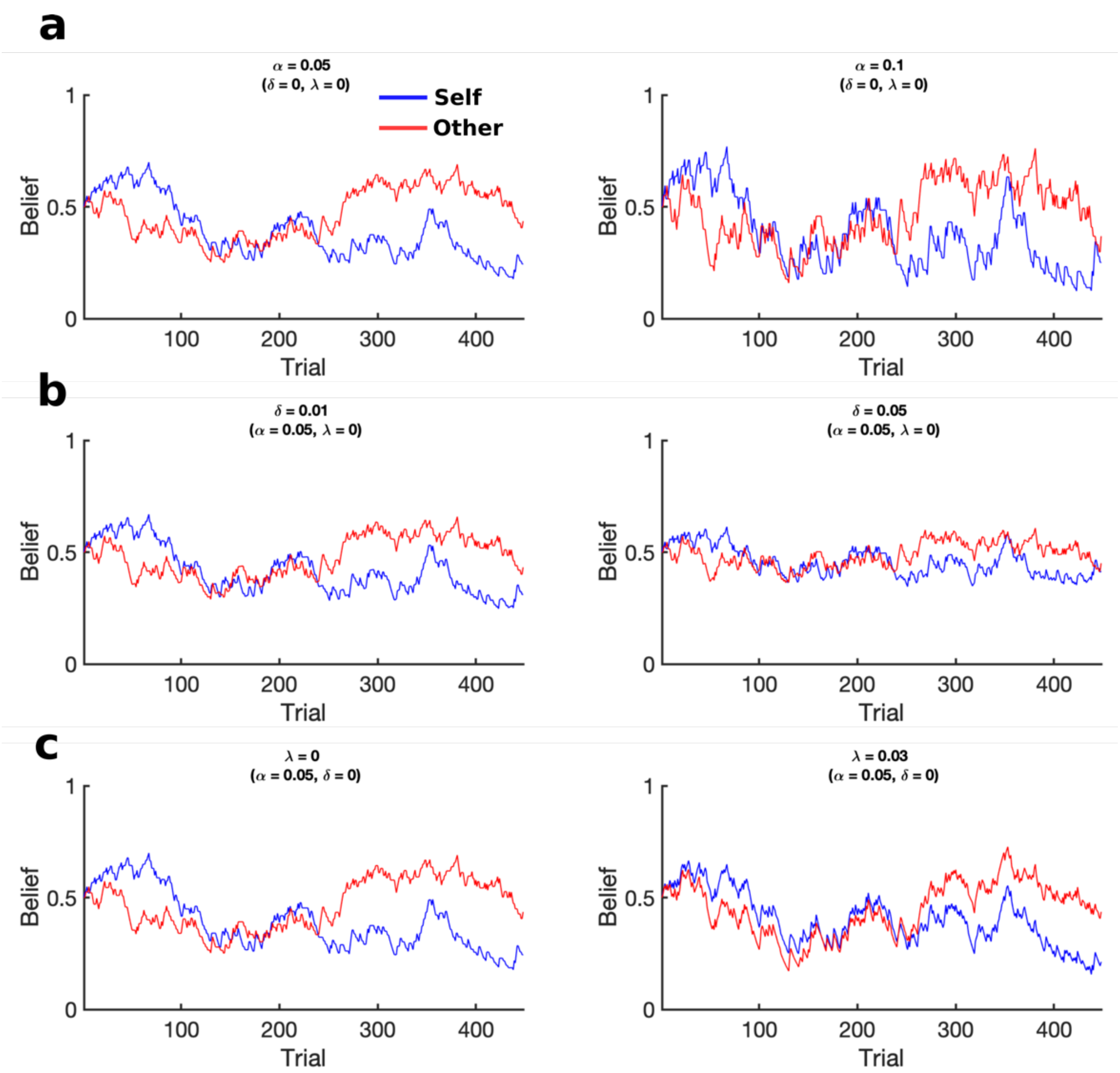

Supplementary Fig. 2 | The functions of the learning parameters. Simulations were used to generate plots to illustrate the effect of varying α, δ and λ in the FBT. **a,** The left plot shows the belief trajectories (attributed to Self and Other) for an example subject with a low learning rate (α). The right plot shows the equivalent with a high α. Increasing α makes the belief trajectories more volatile. **b,** The left plot shows belief trajectories for an example subject with a low memory decay (δ). The right plot shows the equivalent with a high δ. Increasing δ reduces the step size taken along the belief trajectories on each trial, keeping them closer to the starting value of 0.5. **c,** The left plot shows belief trajectories for an example subject with a low leak parameter (λ). The right plot shows the equivalent with a high λ (shared between the two update equations). Increasing λ shifts both belief trajectories so that they are more correlated in time.

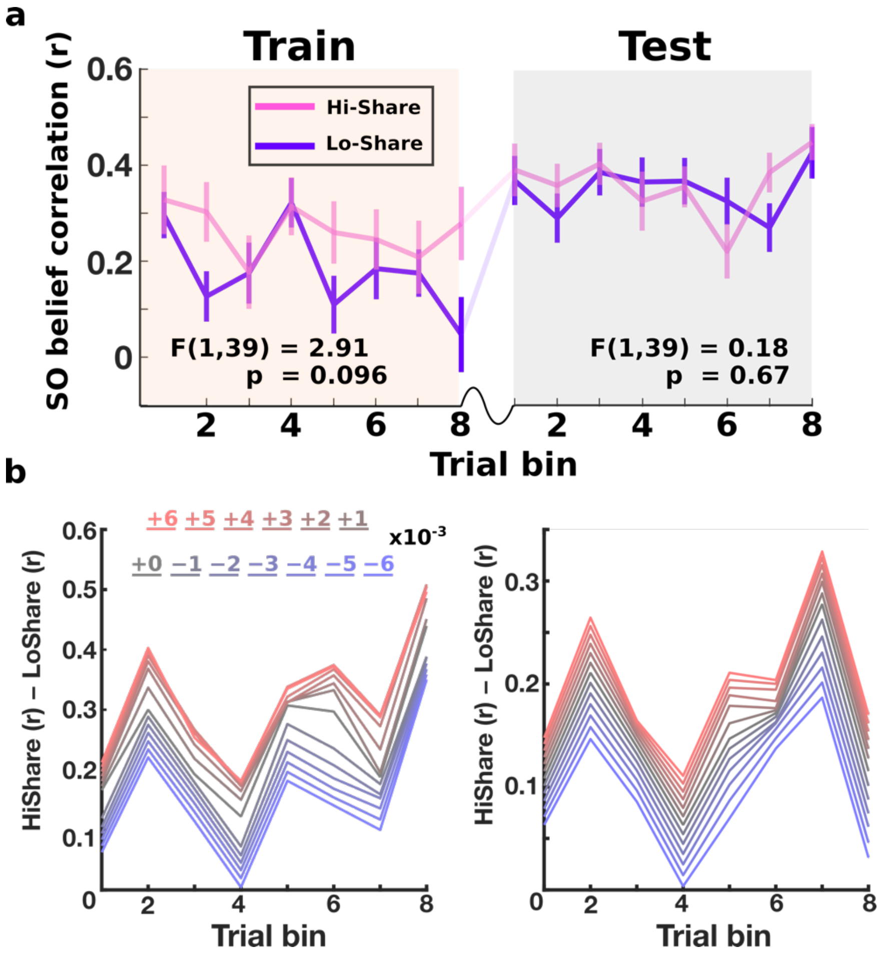

Supplementary Fig. 3 | Simulations show that λ is a determinant of the observed behavioural effects. a, The analysis shown in Fig. 2b was repeated but Self- and Other-attributed beliefs were estimated using the next best-fitting models, that contained no λ parameters. These were models 4 (Lo-Share) and 64 (Hi-Share) for the training session, and model 13 (both conditions) for the testing session. When beliefs were estimated using these models, there was no difference, in training or testing, between the Hi- and Lo-Share conditions, in Self-Other correlation. n = 40 independent subjects. Error bars denote s.e.m. Repeated measures ANOVA testing for main effect of condition in training session $F(1, 39) = 2.91$, $p = 0.096$. Repeated measures ANOVA testing for main effect of condition in testing session $F(1, 39) = 0.18$, $p = 0.67$. **b,** The analysis shown in Fig. 2b was repeated, using the winning models, but each subject's λ parameter in the Hi-Share context was modulated. When the analysis was conducted using increased λ values (red lines), the effect shown in Fig. 2b became stronger. When the analysis was conducted using reduced λ values (blue lines), the effect shown in Fig. 2b became weaker. The range of modulations tested was -0.006 to +0.006. Source data are provided as a Source Data file.

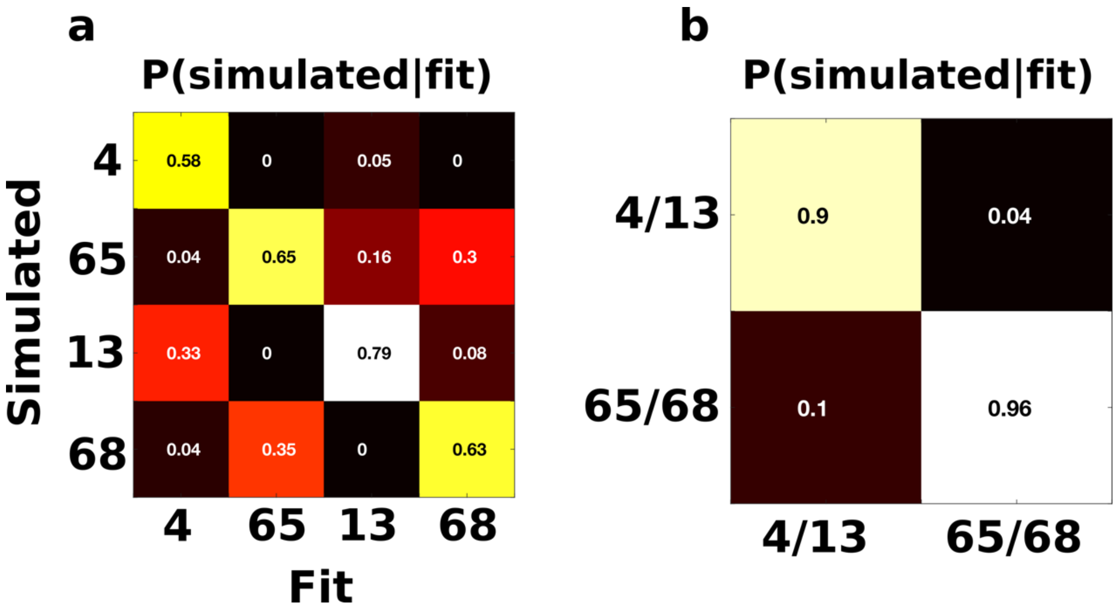

Supplementary Fig. 4 | Model recovery. a, We simulated FBT choice data using each of the 4 best fitting models from for the 4 datasets, using the parameters estimated for each subject. We then fit all 4 models to these simulated data. First, we computed P(fit|simulated) as the probability that a model was the best-fitting of the 4 models, given that it was the generative model. Then, we used Bayes' rule to compute P(simulated|fit) as the probability that a model was the generative model, given that it was the best-fitting of the 4 models. This 'inversion matrix' is shown. For example, the 0.79 in the third row and third column, indicates that if model 13 was the best-fitting, of the four models, then there is a 0.79 chance that this model truly generated the data. See Methods for further details. **b,** We noted that there was a trade-off between models 4 and 13, and also between models 65 and 68. Given that we were primarily interested in being able to distinguish models that contain λ and models that do not contain λ, we repeated the model recovery analysis, but computed the probability that one of the λ-containing models generated the data, given that either of them was the best-fitting model (0.96) and the probability that one of the non-λ-containing models generated the data, given that either of them was the best-fitting model (0.9). This recovery was more successful.

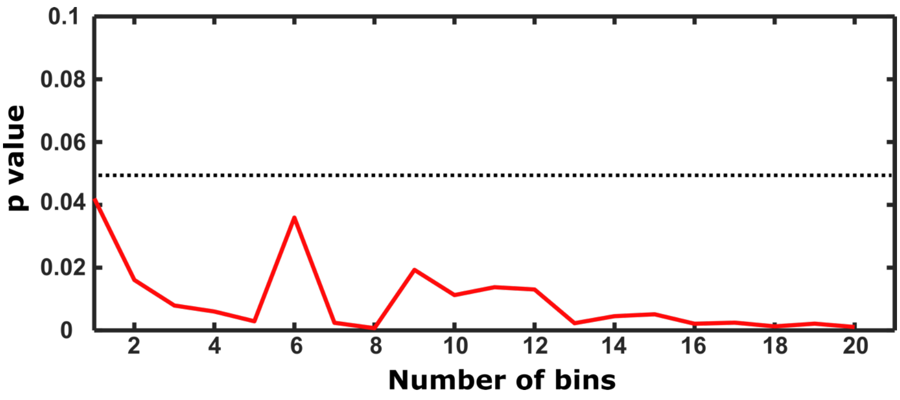

Supplementary Fig. 5 | Main effect on Self-Other correlation with different bin sizes. The analysis shown in Fig. 2b was repeated with different numbers of trial bins. The p-value for the main effect of condition (Lo-Share or Hi-Share) on Self-Other correlation in testing is reported at each number of bins used in the analysis. The p-value is derived from a repeated measures ANOVA $F(1, 39)$. The correlations are significantly higher with the Hi-Share agent than the Lo-Share agent for all bin sizes, showing evidence of correlations at both short and long timescales. Where only one bin was used (i.e. correlation across all trials in the task), the p-value reflects the result of a paired two-sided t-test rather than an ANOVA.

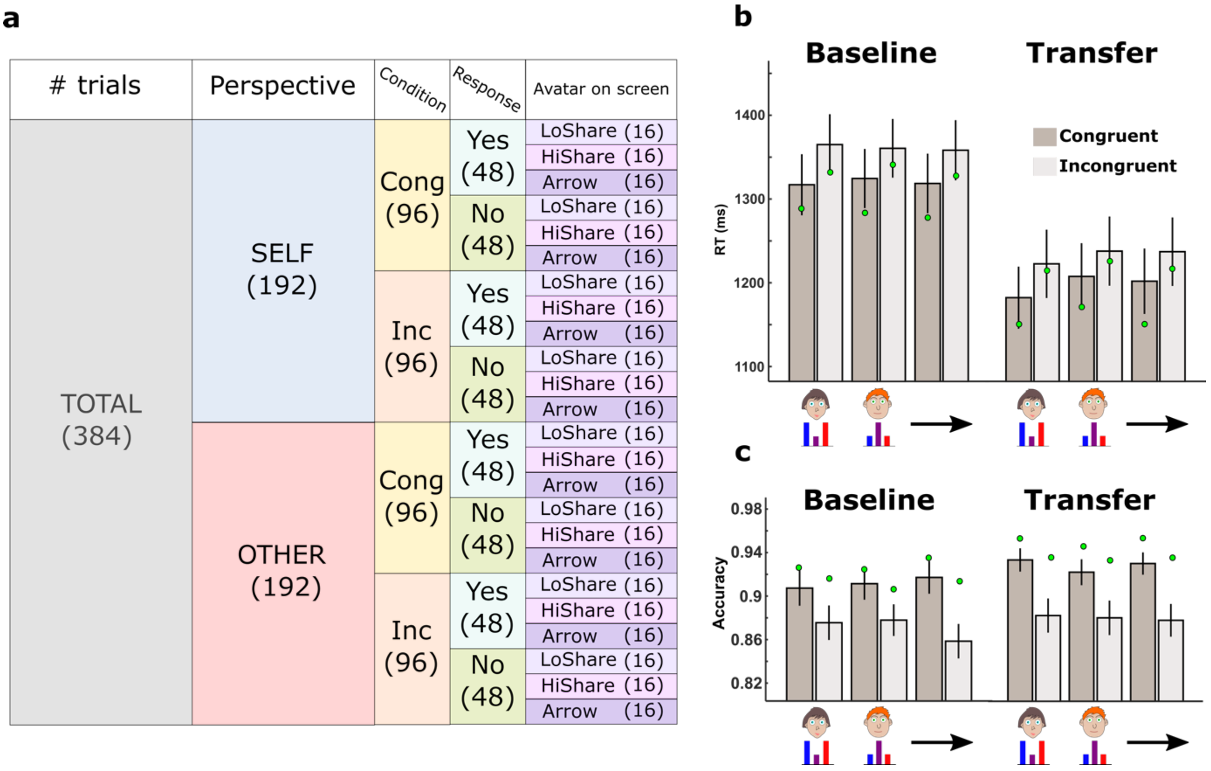

Supplementary Fig. 6 | Factorial design of visual perspective-taking task and empirical data. a, The task consisted of 384 trials in total, presented in a randomised order. Different factors and levels are shown, with the number of trials in each level shown in brackets. For visualisation, the final factor in the task design (direction of avatar eye gaze) is not shown in this table. Cong = congruent. Inc = incongruent. **b,** Response times (RT) at baseline and transfer for different trial types. Self and Other perspective trials have been averaged together, for simplicity. Green circles show simulated data from the drift diffusion model using the median parameters across subjects. **c,** Accuracy, as proportion of correct responses, at baseline and transfer for different trial types. Self and Other perspective trials have been averaged together, for simplicity. Green circles show simulated data from the drift diffusion model using the median parameters across subjects. All error bars denote s.e.m. n = 46 independent subjects. Source data are provided as a Source Data file.

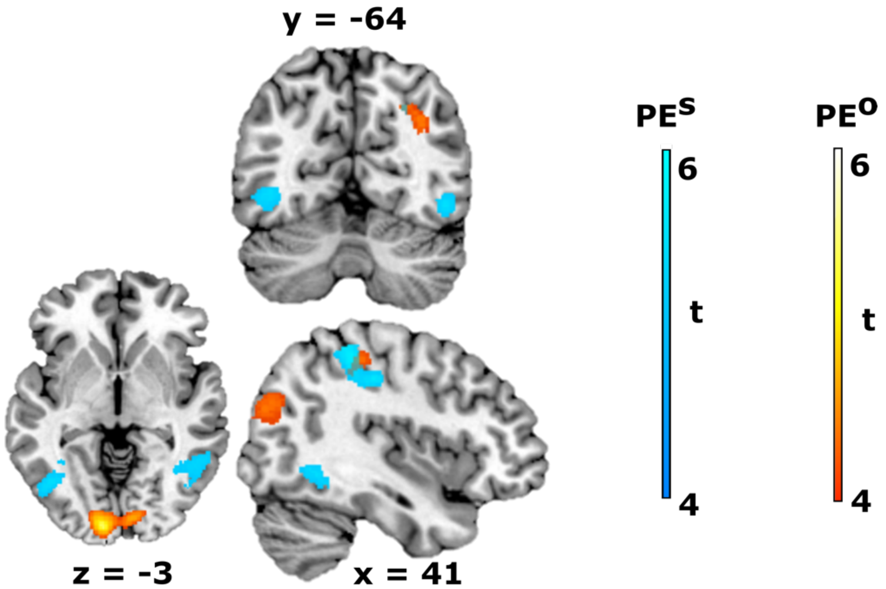

Supplementary Fig. 7 | PE localisation using a mass-univariate approach. Clusters of voxels where BOLD signal covaried with unsigned PE, either Self-attributed (PE^s) or Other-attributed (PE^o), using two one-sided t-contrasts on n = 40 independent subjects. Clusters were defined with a cluster-forming threshold of $p < 0.001$. Only clusters that were large enough to survive FWE-correction at $p < 0.05$ are displayed.

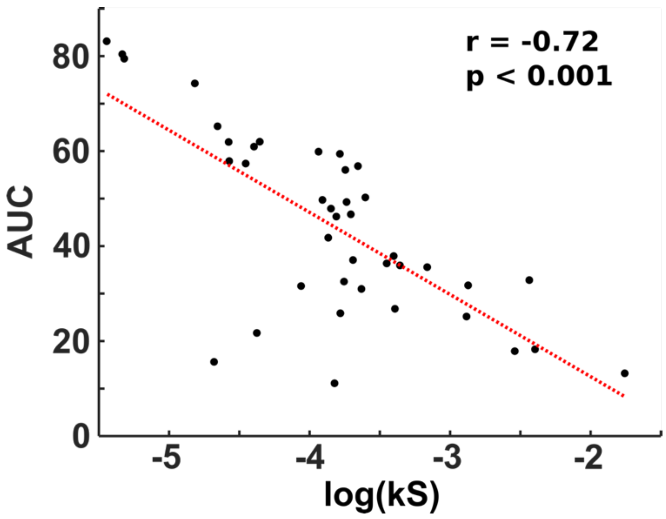

Supplementary Fig. 8 | Validating the model-based discount factor. To ensure that our discount factor log(*kS*) was a reasonable measure of discounting behaviour, we tested whether it was correlated with a model-free measure of discounting behaviour, area under the discounting curve (AUC). Pearson correlation: $r = –0.72$, $p < 0.001$. Source data are provided as a Source Data file.

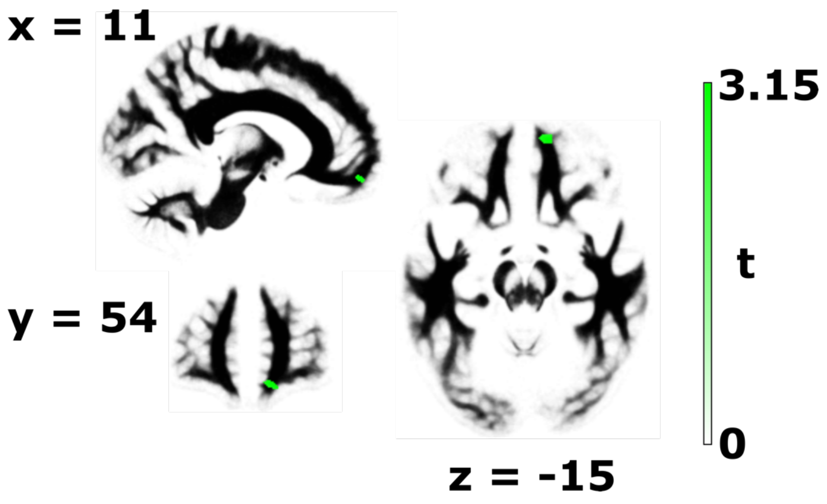

Supplementary Fig. 9 | Ventromedial prefrontal white matter MT is associated with discount factor. Myelin-related MT in a sub-cluster of 201 voxels of vmPFC white matter was negatively associated with discount factor [$p = 0.022$, voxel-level, small-volume corrected].

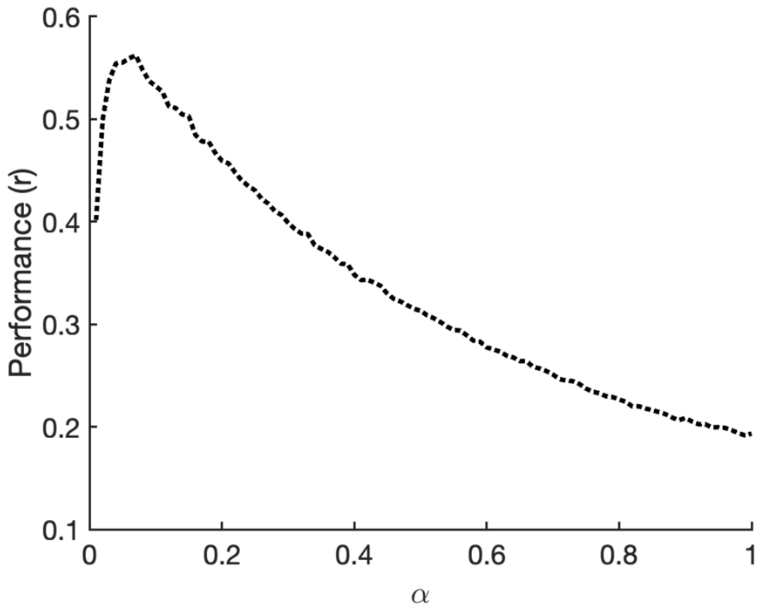

Supplementary Fig. 10 | Optimal learning rate for the FBT. Simulations were used to assess the relationship between task performance on the FBT and learning rate. The optimal learning rate is approximately 0.07.

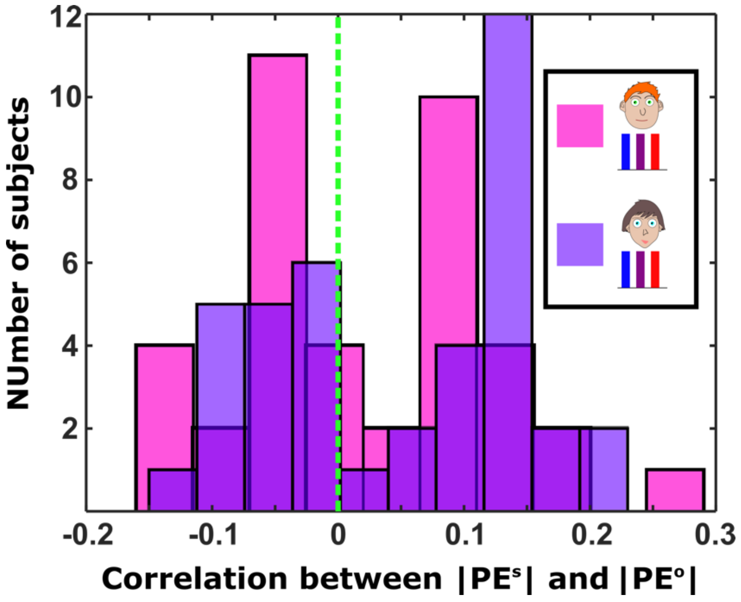

Supplementary Fig. 11 | fMRI regressors were not correlated. For each subject we correlated $|PE^{self}|$ and $|PE^{other}|$ on 'shared' trials. In neither the Lo-Share (purple) or Hi-Share (pink) contexts, did any of the subjects show a significant correlation. n = 40 independent subjects.

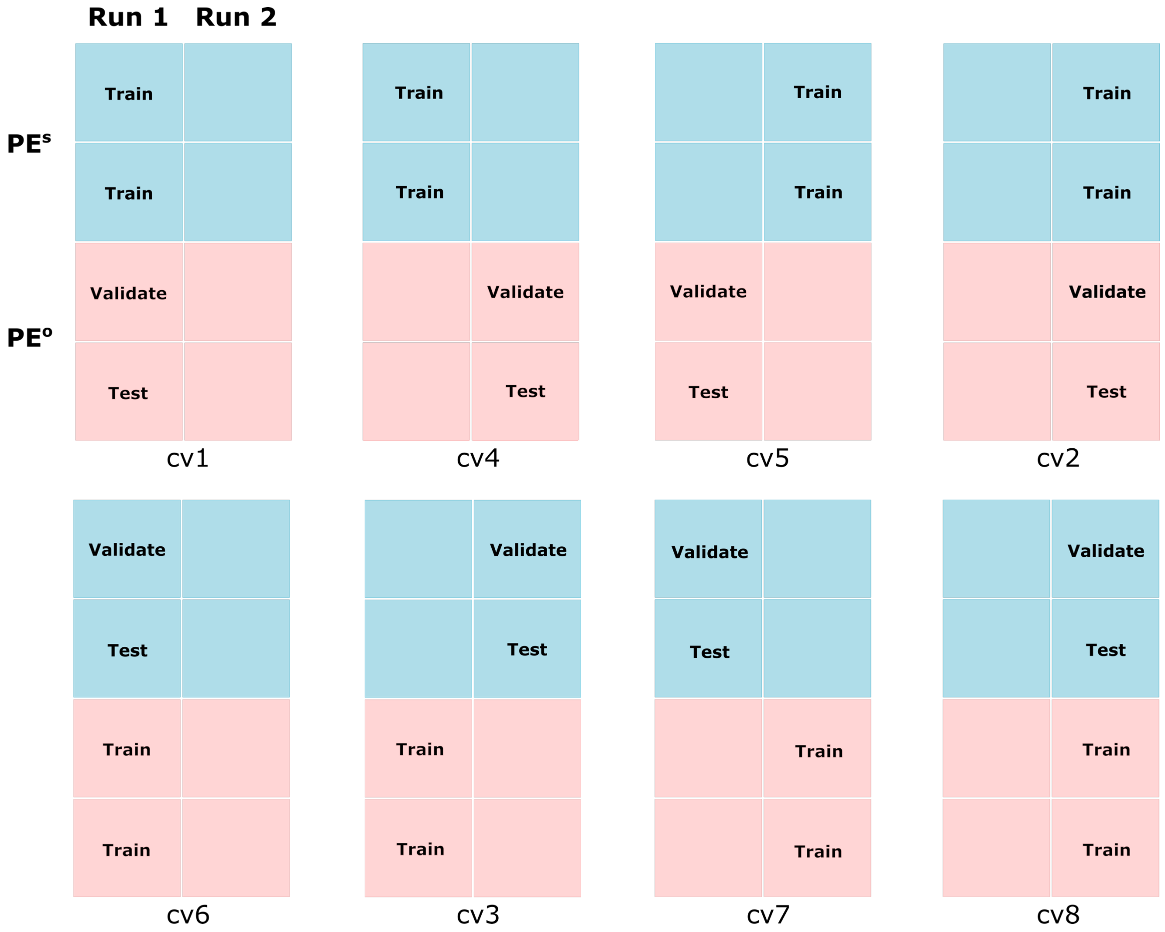

Supplementary Fig. 12 | Cross-validation scheme for cross-decoding analysis. An illustration of how cross-validation was performed for the cross-decoding analysis. For each condition (Hi-Share or Lo-Share) there were two runs of fMRI data. Each run was split into a validation half and a test half. Linear regression models were trained on the $|PE^{\text{self}}|$ or $|PE^{\text{other}}|$ data for one a whole run, validated on half of a run for the alternate signal, and tested on the other half of that same run. Eight folds of cross-validation were carried out in total.

Method	Contrast	P_{FWE} (cluster level)	Cluster size	x	y	z
GLM	PE^s	< 0.001	1835	29	–54	44
GLM	PE^s	< 0.001	981	47	–60	–6
GLM	PE^s	< 0.001	802	–44	–66	–5
GLM	PE^o	<0.001	2365	–9	–93	2
GLM	PE^o	<0.001	1486	36	–77	32
GLM	PE^o	<0.001	723	35	–39	41
GLM	PE^o	0.001	541	–29	–84	26
Searchlight	PE^s	0.018	1632	41	–65	24
Searchlight	PE^s	0.044	1230	29	–53	48
Searchlight	PE^s	0.045	1223	18	–21	54
Searchlight	PE^o	0.02	1586	–18	–92	11

Supplementary table 1 | Summary of significant clusters in PE localisation fMRI analysis. Peak co-ordinates of all significantly large (FWE-corrected $p < 0.05$) clusters identified from one-sided t-contrasts on $n = 40$ independent subjects, using a cluster-forming threshold of $p < 0.001$. Co-ordinates are reported in MNI space. Cluster sizes are reported in number of voxels. Source data is provided as a Source Data file.

ID	α	τ	δ	λ	Total	BIC LO1	BIC HI1	BIC LO2	BIC HI2
M1	1	1	0	0	2	-2956	-2871	-2706	-2912
M2	1	1	0	1	3	-2948	-2819	-2649	-2877
M3	1	1	0	2	4	-2908	-2757	-2609	-2845
M4	1	1	1	0	3	-3044	-2778	-2727	-2928
M5	1	1	1	1	4	-2983	-2710	-2655	-2876
M6	1	1	1	2	5	-2901	-2610	-2613	-2853
M7	1	1	2	0	4	-2940	-2695	-2632	-2860
M8	1	1	2	1	5	-2900	-2678	-2588	-2813
M9	1	1	2	2	6	-2802	-2571	-2530	-2731
M10	1	2	0	0	3	-2950	-3048	-2869	-2980
M11	1	2	0	1	4	-2915	-3001	-2809	-2958
M12	1	2	0	2	5	-2910	-2951	-2771	-2906
M13	1	2	1	0	4	-3036	-2956	-2879	-3004
M14	1	2	1	1	5	-2960	-2857	-2815	-2971
M15	1	2	1	2	6	-2956	-2803	-2736	-2900
M16	1	2	2	0	5	-2930	-2850	-2759	-2916
M17	1	2	2	1	6	-2888	-2791	-2702	-2898
M18	1	2	2	2	7	-2785	-2716	-2636	-2807
M19	2	1	0	0	3	-2923	-2898	-2688	-2873
M20	2	1	0	1	4	-2925	-2871	-2621	-2856
M21	2	1	0	2	5	-2905	-2817	-2590	-2795
M22	2	1	1	0	4	-2983	-2788	-2626	-2860
M23	2	1	1	1	5	-2931	-2734	-2557	-2833
M24	2	1	1	2	6	-2876	-2662	-2560	-2779
M25	2	1	2	0	5	-2854	-2674	-2519	-2779
M26	2	1	2	1	6	-2852	-2671	-2533	-2758
M27	2	1	2	2	7	-2762	-2566	-2432	-2679
M28	2	2	0	0	4	-2903	-3049	-2810	-2932

M29	2	2	0	1	5	-2984	-2997	-2742	-2886
M30	2	2	0	2	6	-2926	-2935	-2680	-2831
M31	2	2	1	0	5	-2975	-2940	-2798	-2955
M32	2	2	1	1	6	-3008	-2866	-2721	-2910
M33	2	2	1	2	7	-2861	-2812	-2646	-2844
M34	2	2	2	0	6	-2847	-2828	-2688	-2843
M35	2	2	2	1	7	-2829	-2770	-2634	-2821
M36	2	2	2	2	8	-2752	-2693	-2564	-2747
M37	2*	1	0	0	3	-2793	-2817	-2558	-2712
M38	2*	1	0	1	4	-2842	-2880	-2561	-2726
M39	2*	1	0	2	5	-2788	-2791	-2571	-2758
M40	2*	1	1	0	4	-2869	-2732	-2557	-2757
M41	2*	1	1	1	5	-2875	-2773	-2554	-2781
M42	2*	1	1	2	6	-2812	-2672	-2559	-2810
M43	2*	1	2	0	5	-2793	-2685	-2521	-2735
M44	2*	1	2	1	6	-2817	-2729	-2555	-2771
M45	2*	1	2	2	7	-2749	-2633	-2583	-2777
M46	2*	2	0	0	4	-2787	-2923	-2728	-2800
M47	2*	2	0	1	5	-2837	-3020	-2717	-2777
M48	2*	2	0	2	6	-2809	-2924	-2698	-2815
M49	2*	2	1	0	5	-2860	-2846	-2724	-2857
M50	2*	2	1	1	6	-2835	-2922	-2734	-2859
M51	2*	2	1	2	7	-2849	-2824	-2696	-2868
M52	2*	2	2	0	6	-2769	-2734	-2642	-2795
M53	2*	2	2	1	7	-2843	-2866	-2659	-2828
M54	2*	2	2	2	8	-2790	-2762	-2623	-2834
M55	4	1	0	0	5	-2875	-2961	-2706	-2812
M56	4	1	0	1	6	-2921	-2995	-2728	-2868
M57	4	1	0	2	7	-2880	-2927	-2731	-2840
M58	4	1	1	0	6	-2916	-2864	-2665	-2827

M59	4	1	1	1	7	-2917	-2893	-2647	-2926
M60	4	1	1	2	8	-2891	-2807	-2654	-2900
M61	4	1	2	0	7	-2786	-2760	-2589	-2769
M62	4	1	2	1	8	-2862	-2783	-2640	-2846
M63	4	1	2	2	9	-2789	-2678	-2581	-2819
M64	4	2	0	0	6	-2832	-3058	-2821	-2870
M65	4	2	0	1	7	-2952	-3078	-2842	-2945
M66	4	2	0	2	8	-2897	-3034	-2793	-2871
M67	4	2	1	0	7	-2878	-2955	-2794	-2922
M68	4	2	1	1	8	-2981	-2999	-2802	-3018
M69	4	2	1	2	9	-2932	-2914	-2755	-2975
M70	4	2	2	0	8	-2767	-2850	-2702	-2824
M71	4	2	2	1	9	-2913	-2929	-2757	-2950
M72	4	2	2	2	10	-2830	-2817	-2684	-2906

Supplementary table 2 | List of models fit to FBT data. 72 models were fit to each of the four behavioural datasets. The number of parameters in each model is shown. In the learning rate column, a '2' indicates that there was a Self-learning rate and an Other-learning rate. A '2*' indicates that there was a learning rate for shared trials and a learning rate for privileged/decoy trials. A '4' indicates that there was a learning rate for Self on privileged trials, Other on decoy trials, Self on shared trials and Other on shared trials. For all other parameters, a '1' indicates a parameter that was shared for Self and Other, and a '2' indicates separate parameters for Self and Other. The BIC columns show the summed BIC across all subjects for each of the four datasets. The lowest BIC for each dataset is highlighted in yellow. Source data is provided as a Source Data file.
