# Supplementary material for: Social training reconfigures prediction errors to shape Self-Other boundaries
Source: Nat Commun. 2020 Jun 15;11:3030. doi: 10.1038/s41467-020-16856-8 (PMC7295766; doi:10.1038/s41467-020-16856-8)
Supplement: Supplementary file 3 — Reporting Summary [file 41467_2020_16856_MOESM3_ESM.pdf]

nature research

Corresponding author(s): Sam Ereira
Last updated by author(s): Apr 25, 2020

Reporting Summary

Nature Research wishes to improve the reproducibility of the work that we publish. This form provides structure for consistency and transparency in reporting. For further information on Nature Research policies, see Authors & Referees and the Editorial Policy Checklist.

Statistics

For all statistical analyses, confirm that the following items are present in the figure legend, table legend, main text, or Methods section.

n/a	Confirmed	
☐	☒	The exact sample size (*n*) for each experimental group/condition, given as a discrete number and unit of measurement
☐	☒	A statement on whether measurements were taken from distinct samples or whether the same sample was measured repeatedly
☐	☒	The statistical test(s) used AND whether they are one- or two-sided<br>*Only common tests should be described solely by name; describe more complex techniques in the Methods section.*
☐	☒	A description of all covariates tested
☐	☒	A description of any assumptions or corrections, such as tests of normality and adjustment for multiple comparisons
☐	☒	A full description of the statistical parameters including central tendency (e.g. means) or other basic estimates (e.g. regression coefficient) AND variation (e.g. standard deviation) or associated estimates of uncertainty (e.g. confidence intervals)
☐	☒	For null hypothesis testing, the test statistic (e.g. *F*, *t*, *r*) with confidence intervals, effect sizes, degrees of freedom and *P* value noted<br>*Give P values as exact values whenever suitable.*
☒	☐	For Bayesian analysis, information on the choice of priors and Markov chain Monte Carlo settings
☒	☐	For hierarchical and complex designs, identification of the appropriate level for tests and full reporting of outcomes
☐	☒	Estimates of effect sizes (e.g. Cohen's *d*, Pearson's *r*), indicating how they were calculated

Our web collection on statistics for biologists contains articles on many of the points above.

Software and code

Policy information about availability of computer code

Data collection	MATLAB R_2018a, Cogent 2000 (v125), Cogent graphics v1.29
Data analysis	MATLAB R_2018a, fast_dm-30.2, SPM12, The Decoding Toolbox v3.994, PhysIO toolbox 7.2.0, hMRI toolbox v0.2.0

For manuscripts utilizing custom algorithms or software that are central to the research but not yet described in published literature, software must be made available to editors/reviewers. We strongly encourage code deposition in a community repository (e.g. GitHub). See the Nature Research guidelines for submitting code & software for further information.

Data

Policy information about availability of data

All manuscripts must include a data availability statement. This statement should provide the following information, where applicable:
- Accession codes, unique identifiers, or web links for publicly available datasets
- A list of figures that have associated raw data
- A description of any restrictions on data availability

All data generated during this study are freely available on the Open Science Framework at [osf.io/62mza/]. The source data underlying Figs. 2a-b, 3b-c, 4b-d, 5c, 6a-b, Supplementary Figures 1, 3a-b, 6b-c and 8 and Supplementary Tables 1 and 2 are also provided as a Source Data file.

Field-specific reporting

Please select the one below that is the best fit for your research. If you are not sure, read the appropriate sections before making your selection.

☒ Life sciences ☐ Behavioural & social sciences ☐ Ecological, evolutionary & environmental sciences

Life sciences study design

All studies must disclose on these points even when the disclosure is negative.

Sample size	We had a pre-determined sample size of 40 participants. We continued to collect data until 40 participants had taken part in the full the study. Our final sample size was 47 because 6 participants did not take part in the full study and 1 participant did not understand the instructions properly. Our pre-determined sample size of 40 was chosen as interindividual variability in task-based computational parameters and decoding accuracies have been previously detected with this sample size before (Ereira et al. 2018). Furthermore, we determined that our microstructural (magnetisation transfer) MRI analysis would require a sample size of 39 in order to detect interindividual variability effects with a power of 90% (see manuscript for more details of power analysis). We therefore deemed a sample size of 40 to be sufficient for our analyses.
Data exclusions	We decided, before data collection started, that data would only be included if a participant understood the behavioural tasks. If a participant did not understand one or more of the behavioural tasks, then we could not be confident that we were indexing the cognitive processes that we intended to, and we would be unable to model the data. Before and after each testing session, participants were asked to report their understanding of the task. Data from 1 participant was entirely excluded because it was evident, upon debriefing, that they did not understand the full task instructions properly.
Replication	Due to local limitations in the availability of resources, the experiment has not yet been repeated to test for reproducibility of findings.
Randomization	The experiment had a within-subjects design. Randomization into groups was not required.
Blinding	Blinding was not necessary as this was a within-subjects design.

Reporting for specific materials, systems and methods

We require information from authors about some types of materials, experimental systems and methods used in many studies. Here, indicate whether each material, system or method listed is relevant to your study. If you are not sure if a list item applies to your research, read the appropriate section before selecting a response.

Materials & experimental systems

n/a	Involved in the study	
☒	☐	Antibodies
☒	☐	Eukaryotic cell lines
☒	☐	Palaeontology
☒	☐	Animals and other organisms
☐	☒	Human research participants
☒	☐	Clinical data

Methods

n/a	Involved in the study	
☒	☐	ChIP-seq
☒	☐	Flow cytometry
☐	☒	MRI-based neuroimaging

Human research participants

Policy information about studies involving human research participants

Population characteristics	47 participants (26 female) aged 19-54. All participants had normal or corrected-to-normal vision and had no history of psychiatric or neurological conditions. There were no other exclusion/inclusion criteria.
Recruitment	Participants were recruited through a local database (ICN subject database) which people can sign up to to hear about research studies. We also advertised through the UCL medical school so that medical students could volunteer to participate. We note that there are self-selection biases in our sample because we only tested participants who approached us with an interest in participating. These participants may have been motivated to take part for financial gain, because of their interest in receiving an MRI scan of their own brain, or perhaps simply due to their interest in cognitive neuroscience. These kinds of subject pools and recruiting strategies are typically used in cognitive neuroscience studies. We can therefore confidently expect our results to generalise to other samples that have been tested within the field, but it is unclear how well these results might generalise to the wider human population, particularly outside of WEIRD societies (white, educated, rich, industrialised, democratic).
Ethics oversight	UCL Research Ethics Committee

Note that full information on the approval of the study protocol must also be provided in the manuscript.

Magnetic resonance imaging

Experimental design

Design type	Task-based. Event-related (parametric) design

Design specifications	4 scanning sessions (runs) per subject. 222 sampling trials per run. Each trial lasted 1.5s with a variable intertrial interval of 1-1.5s. A 'probe' trial was randomly interspersed after every 4-9 sampling trials. A probe trial was a speeded behavioural trial that lasted up to 7 seconds.
Behavioral performance measures	On probe trials we recorded the latency and identity of every button press (moving an arrow left or right along a cursor). We quantified performance by computing a Pearson correlation coefficient between arrow positions and optimal arrow positions.

Acquisition

Imaging type(s)	Functional and Structural
Field strength	3 Tesla
Sequence & imaging parameters	2D EPI functional scans. Each volume comprised 40 slices with a resolution of 3 mm isotropic, with a TR of 2.8 s, TE of 30 ms, slice tilt of –30°, and Z-shim of –0.4. Following the functional scans, a field mapping sequence was used to measure inhomogeneity of the B0 field. This was a double-echo fast low-angle shot (FLASH) sequence with a short TE of 10 ms and a long TE of 12.46 ms. Lastly, a multiple parameter mapping protocol was applied for microstructural imaging. Three 3D multi-echo FLASH acquisitions were made, with predominantly T1, proton density (PD) and magnetisation transfer (MT) weighting respectively. The flip angle was 6° for the PD-weighted and MT-weighted images, and 21° for the T1-weighted images. MT-weighting was achieved through the application of a Gaussian RF pulse 2 kHz off-resonance with 4 ms duration and a nominal flip angle of 220°. The data were acquired with whole-brain coverage at an isotropic resolution of 0.8 mm. Gradient echoes were acquired with alternating readout gradient polarity at eight equidistant echo times ranging from 2.3 to 18.4 ms in steps of 2.3 ms. Only six echoes were acquired for the MT-weighted acquisition in order to maintain a TR of 25 ms of all volumes. Prior to each FLASH acquisition, two additional low resolution (8 mm isotropic) volumes were acquired, one with the 64-channel head and neck array coil and the other with the body coil. A single echo, with a TE of 2.2 ms, was acquired in each case using a 6° flip angle and a TR of 6 ms. The acquisition time of each of these calibration volumes was 5.9 s. These 'sensitivity maps' were used to correct the position-specific modulation of the receive sensitivity field.
Area of acquisition	Whole-brain

Diffusion MRI ☐ Used ☒ Not used

Preprocessing

Preprocessing software	Functional: SPM12. Smoothed with a Gaussian kernel of FWHM 8 mm isotropic.<br>Structural: Quantitative MT maps were created and then spatially processed using the hMRI toolbox v0.2.0 in SPM12. Spatial processing involved three steps: segmentation, diffeomorphic deformation and tissue-weighted smoothing. Each map was converted into grey matter (GM), white matter (WM) and cerebrospinal fluid (CSF) tissue class images. Tissue class images were iteratively aligned a from all of the subjects to their own average before normalising the images to MNI space. Finally, tissue-weighted smoothing was performed with a Gaussian kernel of FWHM 6 mm isotropic. The resulting maps only included those voxels with an a priori probability of being considered in the relevant tissue class (GM, WM or CSF) above 5% and an original tissue density above 5%.
Normalization	Images were normalized into MNI space with default SPM12 settings
Normalization template	MNI
Noise and artifact removal	Motion correction was carried out using the 'realign and unwarp' toolbox within SPM12. Images were co-registered to the first volume acquired for each subject. The motion-corrected images were then unwarped using the field map. Heart rate was monitored using a Nonin 8600FO pulse-oximeter and respiration rate was monitored using a Siemens breathing belt during scanning. Physiological data were converted into 18 nuisance regressors with the PhysIO Toolbox v7.2.0. These 18 regressors and the 6 motion regressors (total of 24 regressors) were included as covariates in 1st level GLMs.
Volume censoring	No volumes were censored

Statistical modeling & inference

Model type and settings	Two separate GLMs were estimated (mass-univariate), one for localising PEself and one for localising PEother. The Self-GLM modelled the onsets of 'privileged' and 'shared' trials, parametrically modulated by |PEself|. The Other-GLM modelled the onsets of 'shared' and 'decoy' trials, parametrically modulated by |PEother|. Temporal and dispersion derivatives were also included. All regressors were z-scored within subjects. The onsets of probes were included in both GLMs, as were 24 nuisance regressors, describing motion and physiological noise. First-level maps were entered into a one-sided t-test at the second level.
Effect(s) tested	T-contrast on |PEself| over privileged and shared trials. T-contrast on |PEother| over shared and decoy trials.

Specify type of analysis: ☒ Whole brain ☐ ROI-based ☐ Both

Statistic type for inference (See Eklund et al. 2016)	Cluster-wise (cluster-forming threshold p<0.001).

Correction	FWE using gaussian random-field theory

Models & analysis

n/a	Involved in the study
☒	☐ Functional and/or effective connectivity
☒	☐ Graph analysis
☐	☒ Multivariate modeling or predictive analysis

Multivariate modeling and predictive analysis

Analysis 1: Whole-brain searchlight analysis. Predicting |PE| magnitude from local BOLD activity in spheres of radius 4 voxels using LASSO linear regression model. Model trained on 3 runs and tested on 4th held-out run. Model evaluated with Fisher z-transformed correlation between model's predicted values and actual |PE| values. L1 penalty was validated against GLM results.

Analysis 2: ROI analysis (approximately 10,000 voxels selected from analysis 1) predicting PE type (self or other) from BOLD activity using LASSO logistic regression. Dimensionality reduction was carried out using principal components analysis. This approach required tuning of two hyperparameters, the L1 penalty and the percentage variance explained by the principal components. We used nested cross-validation to optimise these two hyperparameters. This used a grid-search, sampling over a range of L1 values (10^-5 to 10^-3 in increments of 2.5 x 10^-5) and a range of variance-explained percentages (90%, 92.5%, 95%, 97.5%). Two PE-images from each class were randomly sampled to constitute a hold-out set. The remainder constituted a training set. For each possible pair of hyperparameter values, 40 inner folds of cross-validation were performed on the training set, by randomly sampling two pseudotrials of each class from the training set. Hyperparameters were selected that produced the lowest median cross-entropy across folds. Finally, the classifier with optimised hyperparameters was applied to the original hold-out set, and cross-entropy was measured. This whole procedure was repeated for 40 outer folds of cross-validation and performance was quantified as the median cross-entropy across the 40 outer folds.

Analysis 3: Same as analysis 2 but LASSO linear regression, predicting |PE| magnitude. Regression model was trained on PEself images and tested to make predictions for PEother, and vice versa. The model was evaluated using fisher Z-transformed correlation between true PE magnitude and predicted PE magnitude. In the nested cross-validation there were 8 outer folds and 1 inner fold (see supplementary figure 12).
